# Phenotypic and Genetic Characteristics of *bla*_IMP-6_-Harboring *Enterobacterales* Isolates Lacking *bla*_CTX-M-2_ in Japan

**DOI:** 10.3390/ijms27104269

**Published:** 2026-05-11

**Authors:** Koichi Yamaguchi, Ryuichi Nakano, Akiyo Nakano, Rio Kishi, Kai Saito, Mako Watanabe, Yuki Suzuki, Ryuji Sakata, Miho Ogawa, Hisakazu Yano

**Affiliations:** 1 Department of Microbiology and Infectious Diseases, Nara Medical University, 840 Shijo-cho, Kashihara-shi 6348521, Nara, Japan; ky.5720yy@gmail.com (K.Y.); akiyo@naramed-u.ac.jp (A.N.); suzuki-y@naramed-u.ac.jp (Y.S.); yanohisa@naramed-u.ac.jp (H.Y.); 2 Department of Bacteriology, BML Inc., 1361-1 Matoba, Kawagoe 3501101, Saitama, Japan; sakata-r@bml.co.jp (R.S.); miho-o@bml.co.jp (M.O.)

**Keywords:** antibiotic resistance, carbapenemase, *Enterobacterales*, clinical strains, whole-genome sequencing

## Abstract

Carbapenemase-producing *Enterobacterales* (CPE) is a global threat. IMP-6, a prevalent carbapenemase in western Japan, is mostly disseminated via CTX-M-2 extended-spectrum β-lactamase (ESBL) co-producing *Enterobacterales*. However, the existence and characteristics of *Enterobacterales* harboring *bla*_IMP-6_ without *bla*_CTX-M-2_ remain unclear. We analyzed the phenotypic and genetic characteristics of clinical *bla*_IMP-6_-harboring *Enterobacterales* isolates, focusing on those lacking *bla*_CTX-M-2_. Overall, 220 *bla*_IMP-6_-harboring isolates collected from 76 Japanese hospitals between 2014 and 2021 were characterized by antimicrobial susceptibility, presence of CTX-M-type ESBLs, plasmid incompatibility, plasmid transfer experiments, and genome sequencing and analysis. Among these, 203 co-harbored *bla*_CTX-M-2_ group, with 90% of them demonstrating high conjugation frequency and broad-spectrum resistance to β-lactams. Of the remaining 17 isolates, nine lacked *bla*_CTX-M_, while eight co-harbored *bla*_CTX-M-1_ group (*n* = 2) or *bla*_CTX-M-9_ group (*n* = 6). Eleven isolates carried nontransferable plasmids with genetic structures distinct from those of *bla*_IMP-6_ and *bla*_CTX-M-2_ co-encoding plasmids, including eight non-incompatibility N plasmids. Fifteen isolates carried only *bla*_IMP-6_-encoding plasmids; two carried plasmids with *bla*_IMP-6_ and *bla*_CTX-M_ (*bla*_CTX-M-27_ or *bla*_CTX-M-65_). This novel study revealed that *bla*_IMP-6_ can exist without *bla*_CTX-M-2_ on diverse, often nontransferable plasmids, suggesting distinct, lower dissemination pathways compared to those of epidemic *bla*_CTX-M-2_ co-carrying plasmids and highlighting previously overlooked plasmids that necessitate close monitoring.

## 1. Introduction

*Enterobacterales* are Gram-negative bacteria commonly found in the gastrointestinal tract in healthy individuals; however, it can cause a broad spectrum of severe infections [1,2]. Carbapenemases hydrolyze third-generation cephalosporins and carbapenems [3]. Carbapenemase-producing *Enterobacterales* (CPE) represent an important threat to global health because of limited therapy options and high mortality rates, and CPE outbreaks have been reported worldwide [4,5]. The most frequently detected carbapenemases among *Enterobacterales* are *Klebsiella pneumoniae* carbapenemase (KPC), New Delhi metallo-β-lactamase (NDM), imipenemase (IMP), Verona integron-encoded metallo-β-lactamase (VIM), and oxacillinase (OXA)-48-like enzymes [6]. Carbapenemases are generally categorized into three classes according to the Ambler classification system: Class A (KPC), Class B (NDM, IMP, and VIM), and Class D (OXA-48-like enzymes) [2]. These classes differ with regard to each enzyme’s primary protein structure; specifically, Class B carbapenemases require at least one zinc ion at their active site to facilitate the breakdown of the β-lactam ring [2]. The first IMP-type carbapenemase was found in a clinical *Enterobacterales* isolate from Japan during plasmid-mediated dissemination in 1993, and outbreaks of IMP producers have been reported in not only Asia but also Europe and the Americas [5,7,8,9]. In Japan, CPE are isolated at a significantly low frequency; however, the isolates predominantly harbor IMP-type carbapenemases, particularly IMP-1 or IMP-6 [10,11].

Carbapenemases show regional differences; IMP-1 is predominant in eastern Japan, whereas IMP-6 is dominant in western Japan [10,11]. The first IMP-6-producing *Serratia marcescens* isolate was reported in 1996 [12]. IMP-6-producing *Enterobacterales* isolates have only been reported in Japan [10,11,13,14,15,16]. IMP-6-producing *Pseudomonas aeruginosa* isolates have been reported in South Korea and China [17,18,19,20]. *bla*_IMP-6_ differs from *bla*_IMP-1_ due to an A640G single-base mutation that results in an S214G amino acid substitution. Because of this substitution, the enzymatic activity of IMP-6 for meropenem is significantly higher than that for imipenem, whereas the activity of IMP-1 for meropenem is almost identical to that for imipenem [12,21]. Over 70% of *bla*_IMP-6_-harboring *Escherichia coli* isolates isolated in 2011 were susceptible to not only imipenem but also meropenem [10]; however, *bla*_IMP-6_-harboring *Enterobacterales* can show the inoculum size effect for meropenem [22]. The inoculum size effect is the phenomenon in which the minimum inhibitory concentration (MIC) of an antibiotic increases significantly as the number of bacteria increases. IMP-6 shows weak enzymatic activity to penicillins and monobactams; however, most IMP-6 producers co-harbor cefotaximase-Munich-2 (CTX-M-2) extended-spectrum β-lactamase (ESBL) and consequently show broad-spectrum resistance to β-lactams, including penicillins, third-generation cephalosporins, and monobactams [12,14,15,16,23]. CTX-M-type ESBLs were first reported in the late 1980s and have been recognized as the most globally disseminated ESBL group since the early 2000s [24,25,26]. ESBLs can be clustered into five groups (1, 2, 8, 9, and 25) based on their sequence alignments [24]. Among ESBLs, the CTX-M-9 group, including CTX-M-14 and CTX-M-27, has been isolated mainly in Japan [27,28,29]. However, IMP-6 producers have been reported to encode *bla*_IMP-6_ and *bla*_CTX-M-2_ on plasmids [12,14,15,16,23,28].

Although CPE isolation rates are very low in Japan, the prevalence of *bla*_IMP-6_-harboring *Enterobacterales*, which was associated with the spread of plasmids, was reported in Osaka, western Japan [7,23,30]. Most plasmids were highly homologous to the pKPI-6 plasmid, an incompatibility group N (IncN) plasmid encoding both *bla*_IMP-6_ as the second cassette of a class 1 integron and *bla*_CTX-M-2_ [16], and they showed a high conjugation frequency [7]. IncN plasmids are one of the most frequent broad-host-range plasmids and are regulated by the gene encoding the replicase protein [31]. The first reported isolate encoded *bla*_IMP-6_ on an IncW plasmid [32]; however, the most recently reported clinical isolates harbored *bla*_IMP-6_ and *bla*_CTX-M-2_ encoded on IncN plasmids [16,33]. Although *bla*_IMP-6_, encoded on IncFIA plasmids, was observed among the clinical isolates identified in Osaka [7], there is little information regarding such isolates and other types that may exist.

In this study, we aimed to address the limited understanding about *bla*_IMP-6_-harboring *Enterobacterales* lacking *bla*_CTX-M-2_. We investigated clinical isolates harboring only *bla*_IMP-6_ or co-harboring *bla*_CTX-M_ variants other than *bla*_CTX-M__-2_ and analyzed their phenotypic and genetic characteristics. Through a systematic comparison of *bla*_IMP-6_-harboring *Enterobacterales* lacking *bla*_CTX-M__-2_ with *bla*_CTX-M-2_ co-harboring isolates, we sought to elucidate the differences in antimicrobial susceptibility, plasmid features, and potential dissemination patterns.

## 2. Results

### 2.1. Characteristics of bla_IMP-6_-Harboring Enterobacterales

Among the 220 *bla*_IMP-6_-harboring *Enterobacterales*, 203 co-harbored *bla*_CTX-M-2_ group (*bla*_CTX-M-2_ [*n* = 189], *bla*_CTX-M-35_ [*n* = 11], *bla*_CTX-M-200_ [*n* = 2], *bla*_CTX-M-271_ [*n* = 1]) (Table 1). All 203 isolates were resistant to ampicillin and cefotaxime (Table S1). Regarding carbapenem susceptibility, all 203 isolates were susceptible to imipenem, and a large majority (150/203) were susceptible to meropenem (Table S1). Most isolates (185/203) successfully transferred both *bla*_IMP-6_ and *bla*_CTX-M-2_ group genes to recipient strains via conjugation (Table 1). PCR-based analysis of resistance genes and replicon typing of the transconjugants demonstrated that these genes were co-located on IncN plasmids. Whole-genome sequencing of representative isolates supported the co-encoding of *bla*_IMP-6_ and *bla*_CTX-M-2_ group genes on IncN plasmids further. The mean log_10_ conjugation frequency of these 185 isolates was —3.7 with a 95% confidence interval (CI) of −3.9 to −3.6 (Table S1). All these transconjugants were also resistant to ampicillin and cefotaxime and susceptible to imipenem, whereas 144 were susceptible to meropenem. All 18 nontransferable isolates (*bla*_CTX-M-2_ [*n* = 17], *bla*_CTX-M-200_ [*n* = 1]) carried IncN plasmids.

The other 17 isolates did not co-harbor *bla*_CTX-M-2_; among them, nine did not harbor any *bla*_CTX-M_ genes, while the remaining eight co-harbored *bla*_CTX-M-1_ group (*n* = 2) or *bla*_CTX-M-9_ group (*n* = 6) (Table 1). The antimicrobial susceptibility of the 17 isolates is shown in Table 1. Almost all (16/17) were resistant to ampicillin, and all were resistant to cefotaxime. Regarding carbapenem susceptibility, all were susceptible to imipenem, and a majority (10/17) were susceptible to meropenem. Among the 17 isolates, one non-harboring *bla*_CTX-M_ and five co-harboring *bla*_CTX-M-3_ (*n* = 1) or *bla*_CTX-M-14_ (*n* = 4) transferred only *bla*_IMP-6_-encoding IncN plasmids (Table 1). The mean log_10_ conjugation frequency of these six isolates was −4.3 with a 95% CI of −5.2 to −3.3 (Table 1). All six transconjugants were non-resistant (susceptible or intermediate) to ampicillin, resistant to cefotaxime, and susceptible to imipenem, and five of the transconjugants were non-resistant to meropenem (Table 1). Among the 11 nontransferable *bla*_CTX-M-2_ group non-harboring isolates, only three carried IncN plasmids; the other isolates harbored various incompatibility groups (Table 1). These tendencies were identical for *E. coli*, *K. pneumoniae*, and *Enterobacter cloacae* complex.

### 2.2. Genomic Structures of bla_IMP-6_ and bla_CTX-M-2_ Co-Encoding Plasmids

Whole-genome sequencing analysis was performed on two representative *bla*_CTX-M-2_ co-harboring isolates: transferable NR1442 (biosample accession no. SAMD00898392) and nontransferable NR481 (biosample accession no. SAMD01824540). These isolates were randomly selected from the 203 *bla*_CTX-M-2_ group co-harboring isolates. Both *bla*_CTX-M-2_ group co-encoding IncN plasmids had a conserved, almost complete pKPI-6 locus (accession no. AB616660) regardless of transferability. The Class 1 integron region contained *bla*_IMP-6_, the downstream region of IS*Ecp1* contained *bla*_CTX-M-2_, and the conjugal transfer system of the IncN plasmids was conserved.

### 2.3. Genomic Structures of Only bla_IMP-6_-Encoding Plasmids

**Figure 1:**
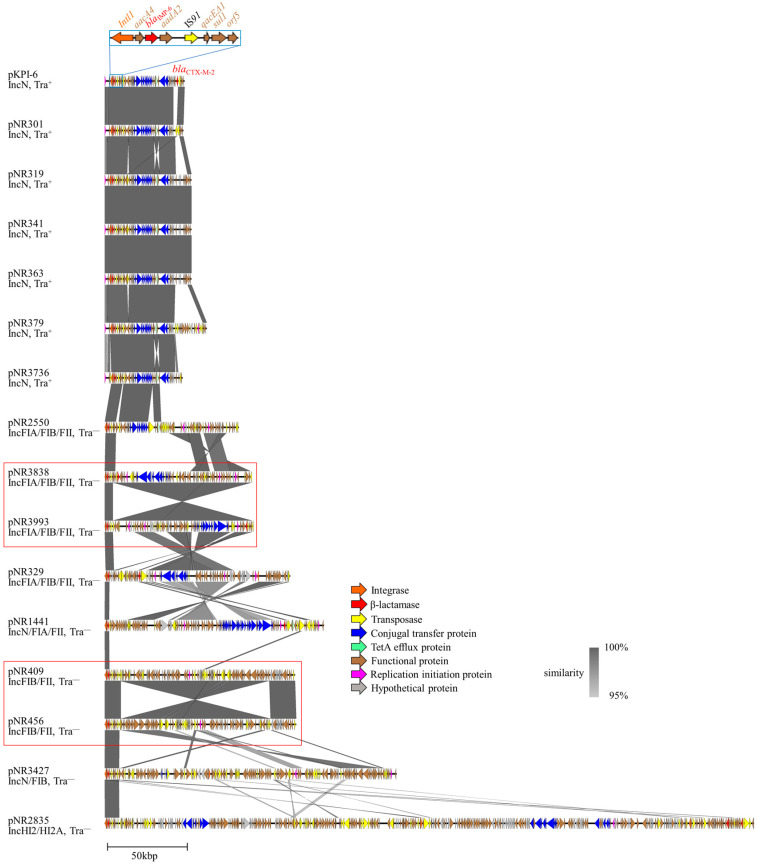
Comparisons of only *bla*_IMP-6_-encoding plasmids with structurally similar plasmids. Linear alignment of only *bla*_IMP-6_-encoding plasmids to a reference plasmid, pKPI-6 (AB616660). See Table S2 in the Supplemental Material for the accession numbers of the *bla*_IMP-6_-encoding plasmids in this study. Blue box: structure of the class 1 integron (In*722*) containing *bla*_IMP-6_ present in all plasmids; red boxes: homologous but partially inverted plasmids. Abbreviations: Tra^+^, transferable; Tra^−^, nontransferable.

Among 17 *bla*_CTX-M-2_ group non-harboring isolates, 15 carried only *bla*_IMP-6_-encoding plasmids (Table S2), while the remaining carried plasmids co-encoding *bla*_IMP-6_ and *bla*_CTX-M_ (*bla*_CTX-M-27_ or *bla*_CTX-M-65_), which were analyzed separately. The accession numbers and characteristics of the 15 only *bla*_IMP-6_-encoding plasmids are summarized in Table S2 as a supplement to Figure 1. Among these 15 isolates, nine did not harbor *bla*_CTX-M_, while the remaining six co-harbored *bla*_CTX-M_ (*bla*_CTX-M-3_ [*n* = 1], *bla*_CTX-M-15_ [*n* = 1], *bla*_CTX-M-14_ [*n* = 4]) on plasmids other than those encoding *bla*_IMP-6_ or chromosome (Table 1). The structural analysis revealed that six transferable plasmids were homologous to pKPI-6, except for the region surrounding *bla*_CTX-M-2_ (Figure 1). Regarding the other nine nontransferable plasmids, the genomic structures of the *bla*_IMP-6_ plasmids and pKPI-6 shared the surrounding region of the class 1 integron containing *bla*_IMP-6_ in common; however, the rest of the genomic structures were diverse (Figure 1). Despite significant differences in genomic structure, the MICs of the isolates harboring only *bla*_IMP-6_ were similar to those of the isolates co-harboring the *bla*_CTX-M-2_ group (Table 1). Inversions occurred on the plasmids between pNR409 and pNR456 and between pNR3838 and pNR3993 (Figure 1). We observed an additional IS*26* element insertion in pNR456 and pNR3993 and IS*26* elements at both ends of the inversion region. Each pair of isolates was collected from different patients in the same hospital at intervals of at least months.

### 2.4. Genomic Structure of the bla_IMP-6_ and bla_CTX-M-27_ Co-Encoding Plasmid

**Figure 2:**
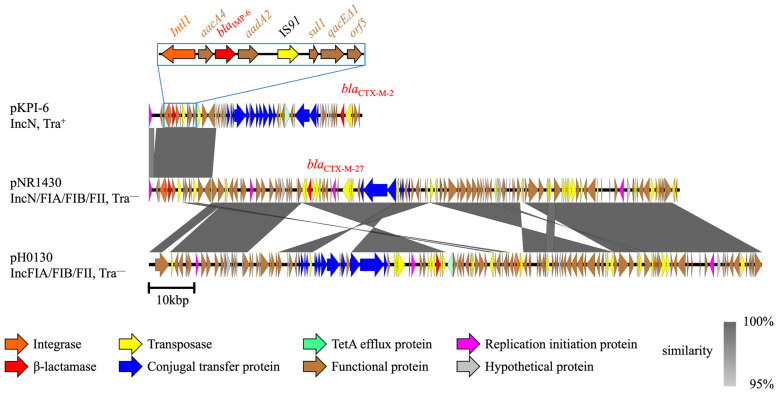
Genetic structure of the *bla*_IMP-6_ and *bla*_CTX-M-27_ co-encoding plasmid pNR1430. Linear alignments of pNR1430 (AP040187) with reference plasmids pKPI-6 (AB616660) and pH0130 (LC520281). Blue box: structure of a class 1 integron (In*722*) containing *bla*_IMP-6_, which is the same as that of pKPI-6. *bla*_CTX-M-27_. Additionally, the surrounding region is similar to that of pH0130. Abbreviations: Tra^+^, transferable; Tra^−^, nontransferable.

Whole-genome sequencing of the nontransferable NR1430 isolate co-harboring *bla*_IMP-6_ and *bla*_CTX-M-27_ revealed that the resistance genes were on a single plasmid (Figure 2). The plasmid and pKPI-6 were IncN plasmids and shared only the region around the class 1 integron (ca. 14 kb), including *bla*_IMP-6_ encoded by pKPI-6. The rest of the genomic structure (ca. 103 kb), including *bla*_CTX-M-27_, differed from that of pKPI-6. Basic local alignment search tool (BLAST) v2.13.0 plasmid sequence alignment showed that the most similar reference plasmid was pH0130 (GenBank accession number LC520281, *E. coli* isolated from Japan in 2016, 90% query coverage, 100% identity). Despite significant differences in genomic structure, the MIC of NR1430 was similar to those of *bla*_CTX-M-2_ group co-harboring isolates (Table 1).

### 2.5. Genomic Structure of the bla_IMP-6_ and bla_CTX-M-65_ Co-Encoding Plasmid

**Figure 3:**
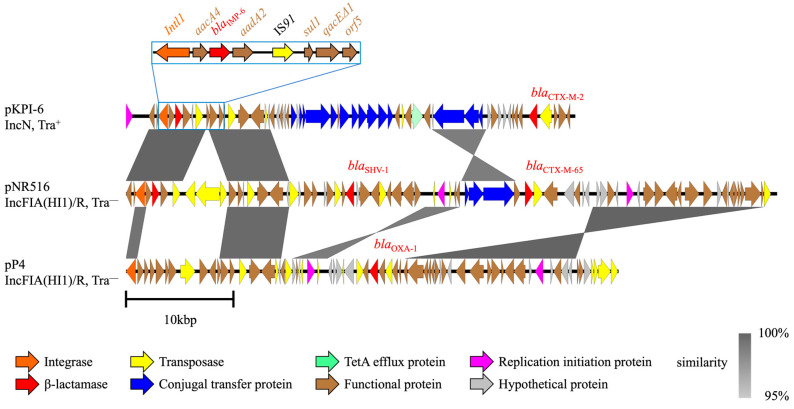
Genetic structure of the *bla*_IMP-6_ and *bla*_CTX-M-65_ co-encoding plasmid pNR516. Linear alignments of pNR516 (AP040228) with reference plasmids pKPI-6 (AB616660) and pP4 (OW968322). Blue box: structure of a class 1 integron (In*722*) containing *bla*_IMP-6_, which is the same as that of pKPI-6. pNR516 has the same genomic content as In*722* except for qacED1. The pP4 reference plasmid is most similar to pNR516. Abbreviations: Tra^+^, transferable; Tra^−^, nontransferable.

Whole-genome sequencing of the nontransferable NR516 isolate harboring *bla*_IMP-6_ and *bla*_CTX-M-65_ revealed that the resistance genes were on a single plasmid (Figure 3). The plasmid was not an IncN plasmid but rather an IncFIA(HI1)/R plasmid; however, the plasmid and pKPI-6 shared a region around the class 1 integron containing *bla*_IMP-6_ and several other genes (ca. 12 kb). The remaining genomic structure (ca. 56 kb), including *bla*_CTX-M-65_, differed from that of pKPI-6, and no homologous *bla*_CTX-M-65_-encoding plasmid was observed. BLAST plasmid sequence alignment showed that the most similar reference plasmid was pP4 (GenBank accession number OW968322, *E. coli* isolated from Spain in 2018, 85% query coverage, 100% identity). Despite the differences in genomic structure, the MIC of NR516 was similar to those of *bla*_CTX-M-2_ group co-harboring isolates (Table 1).

**Table 1:** Characteristics of 220 *bla*_IMP-6_-harboring *Enterobacterales* isolates from 76 hospitals in Japan.

Co-Harboring CTX-M Genes ^a^ (No. of Isolates)		Species ^b^ (Isolates or No. of Isolates)	Donor Isolates ^b^						Transconjugants ^b^										
			MIC or MIC50 (µg/mL)					Incompatibility Group ^f,g^	Conjugation Frequency log_10_ (T/D)			(Transferability)	Co-Harboring CTX-M Genes ^b^	Incompatibility Group of *bla*_IMP-6_ Encoding Plasmids ^g^	MIC or MIC50 (µg/mL) ^h^				
			ABPC	CTX	CAZ	IPM	MEPM		Mean	95%CI					ABPC	CTX	CAZ	IPM	MEPM
CTX-M-2 group (203)	CTX-M-2	EC (95)	>256	128	32	0.25	1	N, others	−3.7	−3.9 to −3.4		(83/95)	CTX-M-2	N	>256	128	16	0.25	0.25
	CTX-M-2	KP (90)	>256	64	32	0.25	1	N, others	−3.8	−4.0 to −3.6		(85/90)	CTX-M-2	N	>256	256	32	0.25	0.5
	CTX-M-2	ECC (4)	>256	256	256	0.5	2	N, others	−3.5	−6.3 to −0.7		(4/4)	CTX-M-2	N	>256	256	256	0.5	2
	CTX-M-35	EC (9)	>256	64	128	0.125	1	N, others	−3.9	−4.2 to −3.5		(9/9)	CTX-M-35	N	>256	32	128	0.25	0.25
	CTX-M-35	KP (NR498)	>256	64	>256	0.125	2	N	−3.5	-	-	(1/1)	CTX-M-35	N	>256	64	>256	0.125	2
	CTX-M-35	KP (NR519)	>256	64	>256	0.125	1	N	−3.7	-	-	(1/1)	CTX-M-35	N	>256	64	>256	0.25	0.5
	CTX-M-200	EC (NR293)	>256	64	32	0.125	0.5	N, FIA, FIB, F	−3.2	-	-	(1/1)	CTX-M-200	N	>256	16	16	0.125	0.5
	CTX-M-200	EC (NR322)	>256	16	8	0.125	0.125	N, FIA, F	nt	-	-	(0/1)	nt	-	-	-	-	-	-
	CTX-M-271	EC (NR3900)	>256	64	64	0.125	4	N, others	−2.5	-	-	(1/1)	CTX-M-271	N	>256	64	8	0.125	4
CTX-M-1 group (2)	CTX-M-3	EC (NR3736)	>256	256	64	0.25	0.5	N, FIB, I1-Iγ, Y, F	−4.4	-	-	(1/1)	-	N	16	32	64	0.5	1
	CTX-M-15	EC (NR1441)	>256	128	128	0.125	2	N, FIA, FII	nt	-	-	(0/1)	nt	-	-	-	-	-	-
CTX-M-9 group (6)	CTX-M-14	EC (NR301)	>256	64	8	0.25	1	N, FIA, F	−5.1	-	-	(1/1)	-	N	64	8	16	0.25	0.25
	CTX-M-14	EC (NR319)	>256	128	32	0.125	0.5	N, FIA, FIB, F	−3.0	-	-	(1/1)	-	N	4	32	32	0.125	0.5
	CTX-M-14	EC (NR341)	>256	128	64	0.5	4	N, FIA, FIB, F	−4.2	-	-	(1/1)	-	N	8	64	32	0.5	4
	CTX-M-14	EC (NR363)	>256	128	128	0.5	4	N, FIA, FIB, F	−3.4	-	-	(1/1)	-	N	16	64	128	0.5	2
	CTX-M-27	EC (NR1430)	>256	64	>256	0.25	1	N, FIA, FIB, FII	nt	-	-	(0/1)	nt	-	-	-	-	-	-
	CTX-M-65	KP (NR516)	>256	>256	32	1	4	FIA(HI1), R	nt	-	-	(0/1)	nt	-	-	-	-	-	-
Non-CTX-M (9)	-	EC (NR379) ^c^	>256	256	>256	0.5	8	N, FIA, A/C	−5.3	-	-	(1/1)	-	N, FIA	4	16	8	0.25	0.25
	-	EC (NR2550)	8	8	8	0.5	8	FIA, FIB, FII	nt	-	-	(0/1)	nt	-	-	-	-	-	-
	-	EC (NR3838)	256	8	8	0.125	0.25	FIA, FIB, FII	nt	-	-	(0/1)	nt	-	-	-	-	-	-
	-	EC (NR3993)	256	4	8	0.125	0.25	FIA, FIB, FII	nt	-	-	(0/1)	nt	-	-	-	-	-	-
	-	EC (NR329)	>256	128	64	0.25	2	FIA, FIB, FII	nt	-	-	(0/1)	nt	-	-	-	-	-	-
	-	KP (NR3427)	32	32	16	0.25	1	N, FIB	nt	-	-	(0/1)	nt	-	-	-	-	-	-
	-	KP (NR409) ^d^	64	16	16	0.125	0.5	FIB, FII	nt	-	-	(0/1)	nt	-	-	-	-	-	-
	-	KP (NR456) ^d^	128	8	16	0.25	0.5	FIB, FII	nt	-	-	(0/1)	nt	-	-	-	-	-	-
	-	ECC (NR2835) ^e^	>256	64	>256	0.5	0.5	HI2, HI2A	nt	-	-	(0/1)	nt	-	-	-	-	-	-

^a^ Underlined co-harboring CTX-M genes are located on plasmids other than those encoding *bla*_IMP-6_ or chromosome. ^b^ Abbreviations: ABPC, ampicillin; CTX, cefotaxime; CAZ, ceftazidime; IPM, imipenem; MEPM, meropenem; EC, *Escherichia coli*; ECC, *Enterobacter cloacae* complex; KP, *Klebsiella pneumoniae*; nt, not transferred; CI, confidence interval; T, initial number of transconjugants; D, initial number of donors. ^c^ Isolates harboring OXA-10. ^d^ Isolates harboring SHV-11. ^e^ Isolates harboring SHV-12. ^f^ Underlined incompatibility groups are *bla*_IMP-6_-encoding plasmids identified using whole-genome sequencing analysis. ^g^ Others include FIA, FIB, 1-Iγ, or Y. ^h^ The MICs for the recipient strain *E. coli* J53 are as follows: AMP, 4 µg/mL; CTX, ≤0.063 µg/mL; CAZ, 0.25 µg/mL; IPM, ≤0.063 µg/mL; and MEM, ≤0.063 µg/mL.

## 3. Discussion

In this study, we provide novel insights into the diversity of *bla*_IMP-6_-harboring *Enterobacterales* lacking *bla*_CTX-M-2_, which have been largely overlooked in previous studies focusing on epidemic *bla*_CTX-M-2_ co-encoding IncN plasmids. Consistent with the results described by previous reports, the majority of the *bla*_IMP-6_-harboring *Enterobacterales* in our nationwide collection co-harbored *bla*_CTX-M-2_ (189/220). In addition, a small subset (*n* = 14) carried variants of the *bla*_CTX-M-2_ group, such as *bla*_CTX-M-35_, *bla*_CTX-M-200_, and *bla*_CTX-M-271_ (accession no. LC844998), which likely originated from *bla*_CTX-M-2_ through single-base mutations (http://www.bldb.eu/ (accessed on 23 February 2025)). Importantly, we identified and characterized a distinct minority population of *bla*_IMP-6_-harboring isolates (*n* = 17) that either lacked *bla*_CTX-M_ or co-harbored *bla*_CTX-M-1_ or *bla*_CTX-M-9_ group genes instead of *bla*_CTX-M-2_, thus highlighting previously underappreciated genetic diversity within this lineage.

Regarding phenotypic characteristics, most isolates (160/220) were susceptible to both imipenem and meropenem regardless of the presence of *bla*_CTX-M_. These isolates were susceptible to carbapenems but might be clinically resistant in vivo because of inoculum size effects [22]. Up to 55.6% of patients with in vitro initial tests showing susceptible KPC-type carbapenemase producers treated with carbapenems experienced clinical or microbiological failure [34], and a similar failure could occur with IMP-6 producers. The European Committee on Antimicrobial Susceptibility Testing (EUCAST) guidelines recommend a MIC screening cut-off of >0.125 mg/L for meropenem and the observation of synergy only with dipicolinic acid for metallo-β-lactamase detection; however, these guidelines included too many applicable isolates. Moreover, three of the isolates might have been overlooked even following the EUCAST recommendation because of the susceptibility of meropenem (MIC ≤ 0.125 mg/L). The detection of such susceptible carbapenemase producers by routine testing using culture-based methods is challenging; therefore, active surveillance is recommended for patients at high risk of carrying carbapenemase producers, including those undergoing major surgery, requiring invasive medical devices, or being treated in environments with suboptimal hand hygiene compliance [23]. A loop-mediated isothermal amplification method and amplification refractory mutation system PCR to distinguish *bla*_IMP-6_ from *bla*_IMP-1_ probably play an important role in rapid and highly sensitive detection of the producers [35,36]. Regarding *bla*_CTX-M_ genes co-harboring isolates, most isolates (154/211) were susceptible to meropenem, and all showed broad-spectrum resistance to β-lactams. Similarly, most of the isolates harboring only *bla*_IMP-6_ (6/9) were susceptible to meropenem; however, all six showed broad-spectrum resistance to β-lactams. Although 10 of 19 isolates subjected to genetic structure analysis harbored *ardK*, which represses *bla*_IMP-6_ transcription by binding the regulatory element [37], no regulatory element was located upstream of *bla*_IMP-6_. Therefore, the meropenem susceptibility of these isolates seemed to have no relationship with *ardK*.

Among the 203 isolates co-harboring the *bla*_CTX-M-2_ group, 185 were transferable with high conjugation frequency, as the *bla*_IMP-6_ and *bla*_CTX-M-2_ groups were encoded on IncN plasmids. The high conjugation frequency of the plasmids and broad-spectrum resistance were consistent with previously reported features of *bla*_CTX-M-2_ group co-harboring isolates [7,14,16,23]. These features were presumed to contribute to the high isolation rate of *bla*_CTX-M-2_ group co-harboring isolates in this study and its dissemination across species. However, even though they carried IncN plasmids, 18 isolates did not transfer. Analysis of the genetic structure of one nontransferable isolate (NR481) revealed that its plasmid was highly homologous to that of one transferable isolate (NR1442). This suggests that chromosomal factors may influence the loss of transferability. However, as we analyzed the genetic structure of only one nontransferable isolate, further comprehensive research is needed to fully elucidate these mechanisms. In contrast, most *bla*_CTX-M-2_ group non-harboring isolates harbored *bla*_IMP-6_ on nontransferable plasmids (11/17), suggesting that the plasmids were less disseminated than *bla*_CTX-M-2_ group co-encoding plasmids. The nontransferable plasmids must explain the low isolation rate of *bla*_CTX-M-2_ group non-harboring isolates in this study. During the prevalence study in Osaka, *bla*_CTX-M-2_ non-harboring isolates were detected [7]. However, unlike the isolates in the current study, these isolates were transferable.

Regarding genetic characteristics, we evaluated the genomic structural similarities and differences between *bla*_CTX-M-2_ co-encoding and non-encoding plasmids. We revealed that while all 19 analyzed plasmids shared a conserved integron structure, the dissemination of *bla*_IMP-6_ is driven by two distinct trajectories: its widespread expansion via a highly conserved IncN plasmid and its simultaneous mobilization into diverse nontransferable plasmids (such as IncF) (Table 2). For the 19 analyzed plasmids, all *bla*_IMP-6_ were encoded as the second cassette of the class 1 integron on plasmids (Table 2), similarly to pKPI-6. Furthermore, all plasmids encoded a derivative of *aacA4* as the first cassette of the class 1 integron and *aadA2* as the third cassette of the class 1 integron, similarly to pKPI-6. In contrast, the plasmid of the original *bla*_IMP-6_-harboring isolate encoded *bla*_IMP-6_ as the first cassette and a hypothetical protein as the second cassette of class 1 integron [12]. Thus, the plasmids in this study and those of the original isolate probably originated from different evolutionary pathways. IMP-6-producing *Pseudomonas aeruginosa* isolates reported in South Korea encoded *bla*_IMP-6_ as the first cassette [20]; therefore, there appears to be no relation between isolates in this study and the previously reported isolates.

**Figure 4:**
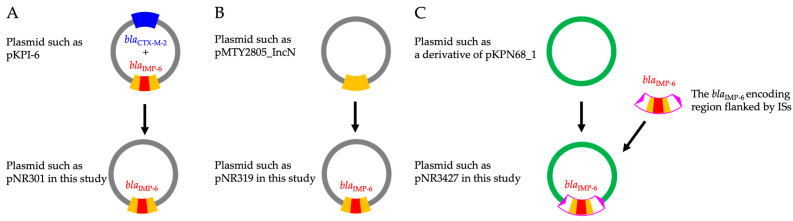
Proposed evolutionary pathways of only *bla*_IMP-6_-encoding plasmids. Schematic representations illustrate the hypothesized transitions of genetic structures. The orange box indicates the integron structure. The pink box indicates the region flanked by ISs, and the pink triangles indicate ISs. (**A**) Proposed pathway of an only *bla*_IMP-6_-encoding plasmid via the subsequent deletion of the *bla*_CTX-M-2_ region, assuming a *bla*_CTX-M-2_ co-encoding plasmid as an ancestor (representative comparison between pNR301 and pKPI-6: 96% query coverage, 99.88% identity). (**B**) Proposed pathway of an only *bla*_IMP-6_-encoding plasmid via insertion of a *bla*_IMP-6_-carrying gene cassette into a class 1 integron, assuming a *bla*_CTX-M_ non-encoding plasmid as an ancestor (representative comparison between pNR319 and pMTY2805_IncN (AP026533): 86% query coverage, 99.97% identity). (**C**) Proposed pathways and only *bla*_IMP-6_-encoding plasmid via insertion of the region flanked by ISs. The other region was highly homologous to a *bla*_CTX-M_ gene non-encoding plasmid (representative comparison between pNR3427 and pKPN68_1 (CP128681): 100% query coverage, 99.99% identity). Abbreviations: IS, insertion sequence.

The two analyzed *bla*_CTX-M-2_ co-encoding IncN plasmids were similar to pKPI-6, including the region surrounding *bla*_IMP-6_ and the conjugal transfer system regardless of transferability (Table 2). Therefore, the homologous conjugal transfer system appears to provide the necessary conditions for the high isolation rate of *bla*_CTX-M-2_ group co-harboring isolates and the high dissemination risk in this study. Among the 17 *bla*_CTX-M-2_ group non-encoding plasmids, six transferable plasmids encoded only *bla*_IMP-6_ on IncN plasmids. The plasmids were highly homologous to pKPI-6, except for the region surrounding *bla*_CTX-M-2_ [16], suggesting that the plasmids were likely disseminated via conjugation. It is difficult to determine the transferable plasmid that came first—*bla*_CTX-M-2_ co-encoding (Figure 4A) or only *bla*_IMP-6_-encoding (Figure 4B)—because some plasmids with genomic structures where *bla*_CTX-M-2_ can be inserted had *bla*_IMP-6_, while others did not [38].

Furthermore, this is the first study to demonstrate the diversity of genomic structures and replicon types among nontransferable *bla*_CTX-M-2_ group non-encoding plasmids (only *bla*_IMP-6_ [*n* = 9], *bla*_CTX-M-27_ [*n* = 1], *bla*_CTX-M-65_ [*n* = 1]) (Table 2). The nine nontransferable only *bla*_IMP-6_-encoding plasmids showed different genomic structures and sizes (Table S3). Moreover, the replicon types were classified into seven groups (Table S3), suggesting that each plasmid was independently derived. Two pairs with the same replicon type (NR409 and NR456, and NR3838 and NR3993) demonstrated an inversion, probably caused by intramolecular IS*26* transposition (Figure 1) [39]. Moreover, using the BLAST database, we identified the reference plasmids with high similarity to each nontransferable *bla*_IMP-6_-encoding plasmid (Table S3). Comparison with the reference plasmids suggests three evolutionary pathways of only *bla*_IMP-6_-encoding plasmids. The first pathway involves deletion of the *bla*_CTX-M-2_ region from a *bla*_CTX-M-2_ co-encoding plasmid such as pKPI-6 (Figure 4A). The second pathway involves the integration of *bla*_IMP-6_ into an existing class 1 integron structure (Figure 4B). This scenario is supported by isolates where nonhomologous regions relative to the reference plasmid were confined within the integron structure. The third pathway involves the insertion of a larger genetic module region harboring *bla*_IMP-6_, as a transposon flanked by insertion sequence (IS) elements, into an existing plasmid (Figure 4C). This pathway is considered unique and different from the one proposed for *bla*_CTX-M-2_ co-encoding plasmids, as evidenced by cases where the non-homologous regions extended beyond the integron structure into the adjacent surrounding sequences. Two of the reference plasmids encoded *bla*_IMP-6_ as the second cassette of the class 1 integron (Table S3). However, no reference plasmid was available to suggest a base substitution between *bla*_IMP-6_ and *bla*_IMP-1_ as for the relationship between pKPI-6, which encodes *bla*_IMP-6_, and pKPI-1, which encodes *bla*_IMP-1_ (96% query coverage, 100% identity). Some *bla*_CTX-M-2_ group non-encoding plasmids isolated during the prevalence study in Osaka showed high similarity to each other [7], and the most similar plasmid in this study to those plasmids was pNR1441 (80% query coverage, 99.98% identity).

In addition, we newly characterized plasmids co-encoding *bla*_IMP-6_ and *bla*_CTX-M_ (*bla*_CTX-M-27_ or *bla*_CTX-M-65_). The genetic structure of *bla*_IMP-6_ and *bla*_CTX-M-27_ co-encoding IncN plasmid was homologous to pH0130 except for the region surrounding *bla*_IMP-6_, including *bla*_CTX-M-27_ (Figure 2). The region surrounding *bla*_IMP-6_ flanked by ISs was likely to be inserted into a derivative of pH0130; however, the mechanism is unknown. The region surrounding *bla*_IMP-6_ flanked by ISs encompasses not only the class 1 integron but also additional genes that are identical to those found in *bla*_CTX-M-2_ co-encoding plasmids (Figure 2). Given that the *bla*_CTX-M-27_ co-encoding plasmid harbors this conserved region, it is highly probable that the plasmid emerged subsequent to *bla*_CTX-M-2_ co-encoding plasmids, likely through acquisition of this conserved region. The model is likely the same as that shown in Figure 4C. H0130 and the isolate with an unknown genetic structure that co-harbors *bla*_IMP-6_ and *bla*_CTX-M-27_ have been isolated in Japan [15,40]. Furthermore, CTX-M-27 confers a high MIC for ceftazidime [41], consistent with the features of the *bla*_IMP-6_ and *bla*_CTX-M-27_ co-harboring isolate in this study (Table 1). Moreover, CTX-M-27 is the predominant CTX-M subtype of ESBL isolated in Japan [28,29]. These characteristics of CTX-M-27 appear to involve the emergence of *bla*_IMP-6_ and *bla*_CTX-M-27_ co-encoding plasmids; therefore, continuous monitoring of these plasmids is needed. Similarly, we reported the genomic structure of the *bla*_IMP-6_ and *bla*_CTX-M-65_ co-encoding IncFIA(HI1)/R plasmid. Regarding the genomic structure, except for the region surrounding *bla*_IMP-6_, including *bla*_CTX-M-65_ (Figure 3), we observed no homologous *bla*_CTX-M-65_ encoding plasmid, indicating that the plasmid was newly emerged.

This study presents some limitations. First, it was conducted using a limited variety of isolates from Japan. To assess the characteristics of *bla*_IMP-6_-harboring *Enterobacterales*, global strains must be evaluated. Second, we performed genome sequencing and analysis for only two of 203 *Enterobacterales* isolates co-harboring *bla*_IMP-6_ and *bla*_CTX-M-2_. With regard to the genetic structure of plasmids, the remaining isolates may have structures that are different from those of these two isolates; however, the isolates probably had the same structure because they showed high conjugation frequency given that they were encoded on IncN plasmids. In contrast, other genetic components such as chromosomal backgrounds and endogenous resistance genes likely harbor variations among individual isolates. A comprehensive whole-genome sequencing analysis of the entire cohort would be required for accurate characterization of these features.

## 4. Materials and Methods

### 4.1. Bacterial Isolates and Antibacterial Susceptibility Testing

A total of 220 non-redundant, clinical *bla*_IMP-6_-harboring *Enterobacterales* isolates, including 119 *E. coli* isolates, 96 *K. pneumoniae* isolates, and five *E. cloacae* complex isolates, were collected from 76 hospitals in Japan from 2014 to 2021 (Table S1). Species identification for each isolate was verified using the MicroScan WalkAway system (Siemens Healthineers Diagnostics, Tokyo, Japan), MALDI Biotyper (Bruker Daltonics, Billerica, MA, USA), or VITEK (bioMérieux, Marcy l’Étoile, France) at each hospital. All isolates were screened on the basis of a ceftazidime MIC ≥ 8 μg/mL using the MicroScan WalkAway system, VITEK, DPS192iX (Eiken Chemical Co., Ltd., Tokyo, Japan), or IA20MIC mkII (Eiken Chemical Co., Ltd., Tokyo, Japan). The carbapenem inactivation method was used to screen for carbapenemase production as previously described [42]. The MIC of the collected isolates was evaluated using the agar dilution method according to the Clinical and Laboratory Standards Institute guidelines [26]. *E. coli* ATCC 29522 was used for quality control.

### 4.2. Polymerase Chain Reaction (PCR) Identification and Sequencing of β-Lactamase Genes

*bla*_IMP-6_ was detected and confirmed using PCR and DNA sequencing, respectively [35]. The presence of CTX-M-type ESBLs was determined using PCR and sequencing. *bla*_CTX-M_ genes were detected using PCR with CTX-M-1, CTX-M-2, CTX-M-8, CTX-M-9, and CTX-M-25 group-specific primers [43]. The amplified PCR products were confirmed using DNA sequencing. Sequence alignment and analysis were performed using BLAST (https://blast.ncbi.nlm.nih.gov/Blast.cgi (accessed on 20 June 2022)).

### 4.3. Plasmid Transfer Experiment and Replicon Typing

The transferability of 220 *bla*_IMP-6_-harboring *Enterobacterales* isolates was investigated in conjugation experiments using CPE as the donor and sodium azide-resistant *E. coli* J53 as the recipient, as previously described [44]. Luria–Bertani broth cultures of donor strains and the recipient *E. coli* J53 strain at exponential-phase growth were mixed at a ratio of 1:1 by volume and then incubated overnight at 37 °C. Then, we selected transconjugants on Luria–Bertani agar plates containing cefpodoxime (8 µg/mL) and sodium azide (100 µg/mL). The conjugation frequency was expressed as the log_10_ ratio of transconjugants to donors, as described previously [45]. For transferable isolates, the mean conjugation frequency and its 95% CI were calculated based on these log-transformed values. The transfer of resistance genes carried by the recipient strains was verified using PCR. Plasmid incompatibility was identified using PCR-based replicon typing [46].

### 4.4. Genome Sequencing and Analysis

The previously uncharacterized genetic structures of all 17 *bla*_CTX-M-2_ non-harboring isolates were investigated by genome sequencing, as little information about their plasmid structures and genetic backgrounds is available. In contrast, of the *bla*_CTX-M-2_ group co-harboring isolates, two representative isolates were selected for genome sequencing to assess the genomic structural similarities and differences between *bla*_CTX-M-2_ co-encoding and non-encoding plasmids, because *bla*_CTX-M-2_-harboring isolates carry highly conserved IncN plasmids similarly to pKPI-6. The selection was based on the predominant genotype (*bla*_CTX-M-2_) and incompatibility group (IncN), sharing the same characteristics as pKPI-6. For variations in dissemination, one transferable (*E. coli* NR1442) and one nontransferable isolate (*K. pneumoniae* NR481) each were randomly selected from the predominant species: *E. coli* and *K. pneumoniae*. DNA was extracted from each isolate using the magLEAD 6gC (Precision System Science Co., Ltd., Chiba, Japan) and sequenced using MiSeq (Illumina, San Diego, CA, USA), MinION (Oxford Nanopore Technologies, Oxford, UK), and Sanger sequencing. After read trimming and quality filtering, hybrid de novo assemblies of Illumina and Nanopore reads were generated using Unicycler v0.5.0 [47] or QIAGEN CLC Genomics Workbench 24.0 (QIAGEN, Aarhus, Denmark). The assembled sequences were annotated using DFAST v1.6.0 with standard settings [48,49] and the BLAST algorithm. Antimicrobial resistance genes and plasmid replicon types were detected using ResFinder 4.1 [50] and PlasmidFinder 2.1 (https://cge.food.dtu.dk/services/PlasmidFinder/ (accessed on 4 August 2022)), respectively. The genomic structures were compared using Easyfig, v2.2.2.

## 5. Conclusions

In conclusion, our findings revealed the phenotypic and genotypic characteristics of *bla*_IMP-6_-harboring *Enterobacterales* isolated in Japan. With regard to phenotypic characteristics, although *Enterobacterales* harboring *bla*_IMP-6_ without *bla*_CTX-M-2_ were isolated at a low frequency, they existed and mostly carried nontransferable *bla*_IMP-6_-encoding plasmids. With regard to genetic characteristics, to our knowledge, this is the first study to report that the characteristics of *bla*_IMP-6_-harboring *Enterobacterales* without *bla*_CTX-M-2_ exhibited diversity and were different from those of *Enterobacterales* co-harboring *bla*_CTX-M-2_. Furthermore, we identified the genetic structures of plasmids encoding new combinations of *bla*_IMP-6_ and *bla*_CTX-M_ (*bla*_CTX-M-27_ or *bla*_CTX-M-65_). These isolates must have been previously overlooked because of the lower dissemination; therefore, close monitoring is required. As other combinations may exist or appear, continued analyses and further laboratory experiments will provide deeper insights into the evolutionary paths of *bla*_IMP-6_-harboring *Enterobacterales*.

## Figures and Tables

**Table 2:** Phenotypic and genetic characteristics of three types of *bla*_IMP-6_-encoding plasmids.

	*bla*_CTX-M-2_ Group Co-Encoding Plasmids (*n* = 203)	Only *bla*_IMP-6_ Co-Encoding Plasmids (*n* = 15)	*bla*_CTX-M-27_ (*n* = 1) or *bla*_CTX-M-65_ (*n* = 1) Co-Encoding Plasmids
Coding region of *bla*_IMP-6_	The second cassette of class 1 integron		
Transferability	Almost always transferable	Often nontransferable	Nontransferable
Incompatibility group	N	Various replicon types	*bla*_CTX-M-27_: N, FIA, FIB, FII *bla*_CTX-M-65_: FIA(HI1), R
A reference plasmid with a similar genetic structure	pKPI-6	No common reference plasmid	

## Data Availability

The genome sequencing data presented in this study are deposited under BioProject accession number PRJDB20582.

## Supplementary Materials

The following supporting information can be downloaded at: https://www.mdpi.com/article/10.3390/ijms27104269/s1.

## Abbreviations

The following abbreviations are used in this manuscript:

CPE	carbapenemase-producing *Enterobacterales*
KPC	*Klebsiella pneumoniae* carbapenemase
IMP	imipenemase
CTX-M-2	cefotaximase-Munich-2
ESBL	extended-spectrum β-lactamase
IncN	incompatibility group N
MICs	minimum inhibitory concentrations
BLAST	basic local alignment search tool
EUCAST	European Committee on Antimicrobial Susceptibility Testing
PCR	polymerase chain reaction
